# Supplementary material for: Snowprint: a predictive tool for genetic biosensor discovery
Source: Commun Biol. 2024 Feb 9;7:163. doi: 10.1038/s42003-024-05849-8 (PMC10858194; doi:10.1038/s42003-024-05849-8)
Supplement: Supplementary file 2 — Supplementary Information [file 42003_2024_5849_MOESM2_ESM.pdf]

Snowprint: a predictive tool for genetic biosensor discovery

Simon d'Oelsnitz* [1,2], Sarah K. Stofel[1], Joshua D. Love[3], Andrew D. Ellington[1]

Affiliations

[1]Department of Molecular Biosciences, University of Texas at Austin, Austin, TX, 78712, USA

[2]Present address: Department of Systems Biology, Harvard Medical School, Boston MA, 02115, USA

[3]Independent Web Developer, Bentonville, Arkansas 72712, United States

Supplementary Information:

Supplementary Figures 1 - 12

Supplementary Tables 1, 2

Supplementary Notes 1, 2

Supplementary Figures

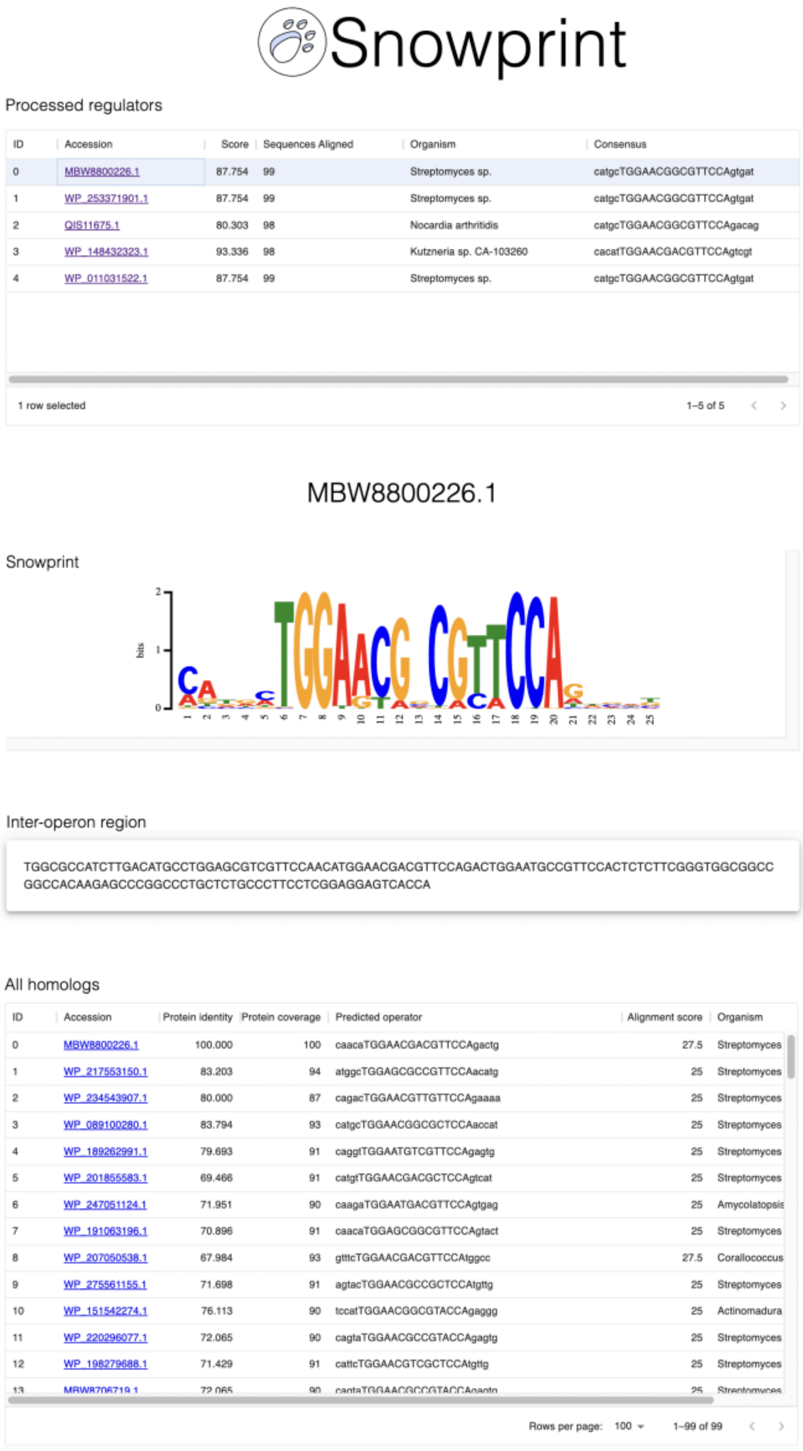

Supplementary Figure 1. React-based local web page displaying results from a Snowprint query.

Users can browse among predictions made for regulators run locally and view the (1) consensus prediction logo, (2) Inter-operon region containing the predicted operator, and (3) homologous predicted operators used to create the consensus prediction displayed in (1). Sequence logos are generated using LogoJS[1]. The Snowprint logo was generated using a vector graphics editor.

a

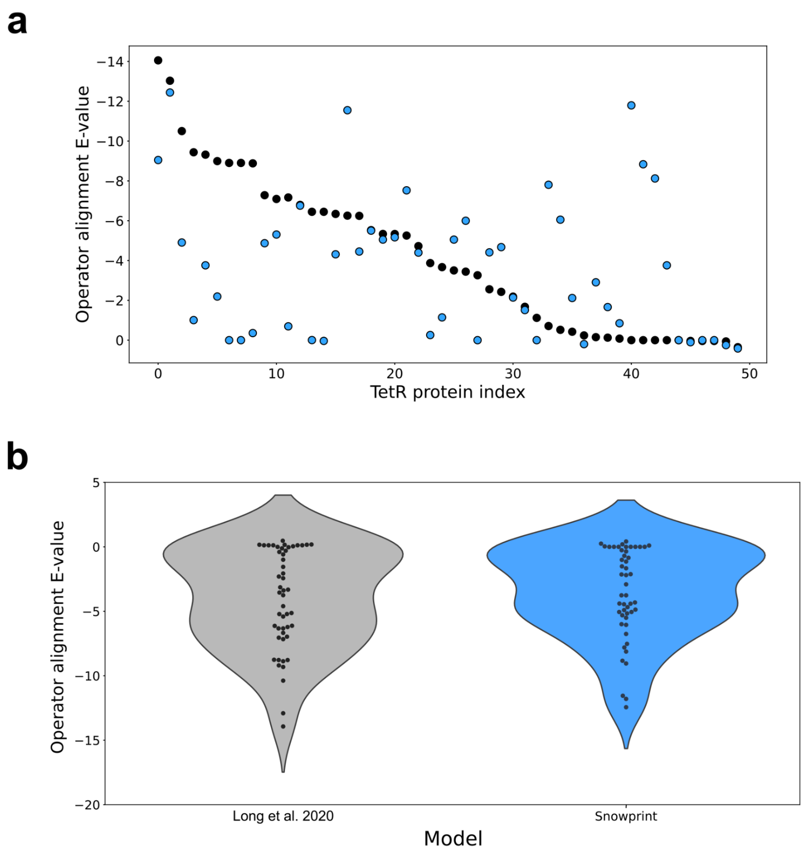

Supplementary Figure 2. Comparison of Snowprint to a published statistical model

Predictions were generated for 50 TetR-family proteins using Snowprint and a statistical model published by Long et al. (a) The E-value of the predicted operator aligned to the experimentally validated operator is plotted for each TetR-family protein. See **Supplementary Data 2** for all model inputs, predicted operators, and E-values used to generate this plot. (b) Violin plots describing the global distribution of validated:predicted operator E-values between the two models. For both (a) and (b) blue represents Snowprint predictions and black represents statistical model predictions.

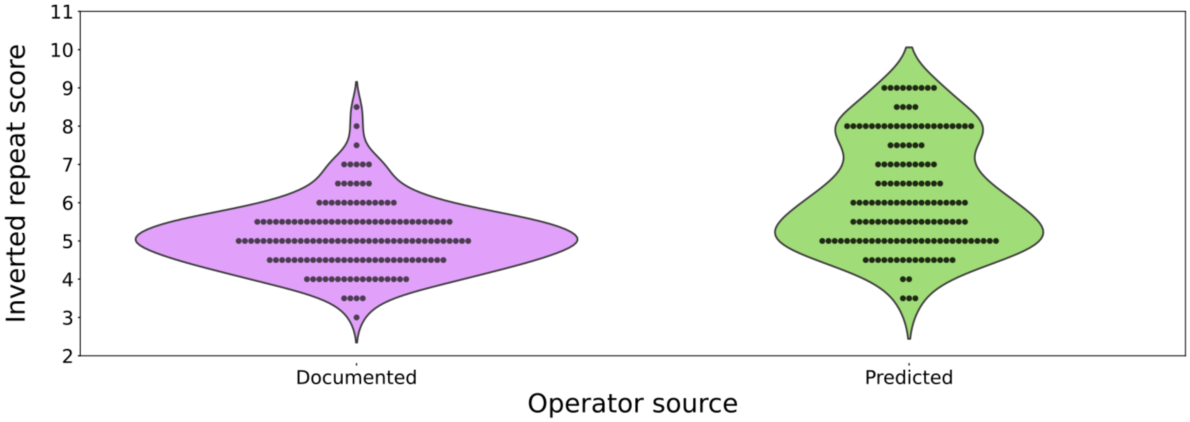

Supplementary Figure 3. Inverted repeat scores of operators within the benchmarking dataset.
Documented operators, shown in purple, represent the operator sequences extracted from literature (see **Supplementary Data 1**), while predicted operators, shown in green, represent operator sequences predicted by Snowprint using the associated regulator RefSeq accession ID.

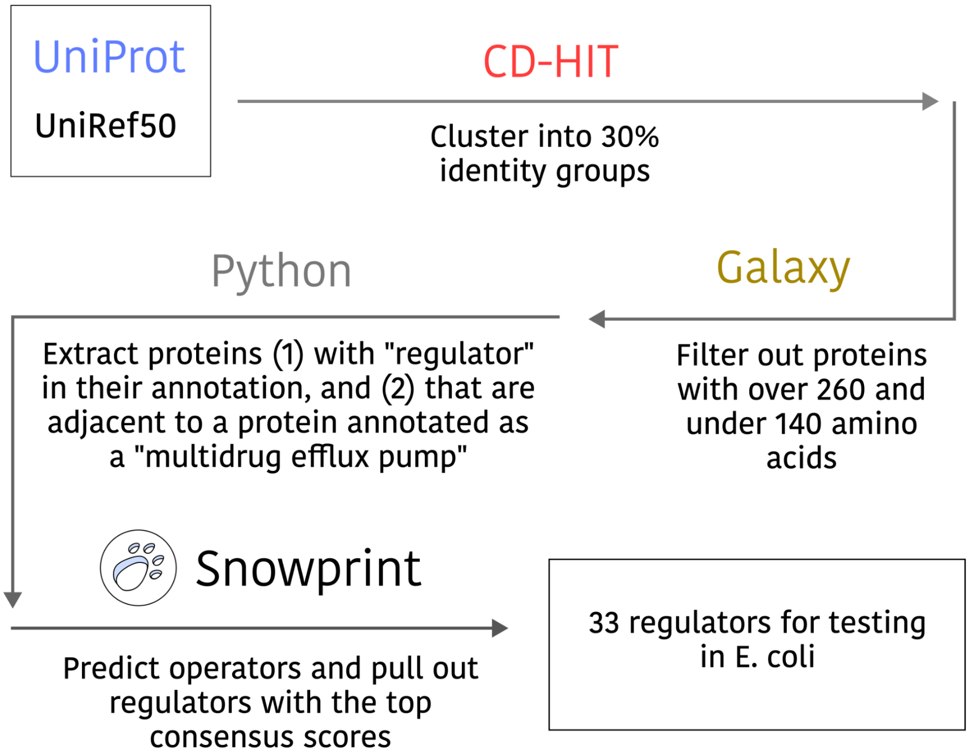

Supplementary Figure 4. Generalist regulator curation workflow.

TetR-family regulators were fetched from the UniRef50 sequence database, which were then clustered into 30% similarity groups using the CD-HIT software and filtered to contain only protein sequences shorter than 260 amino acids and longer than 140 amino acids using the Galaxy Suite software. Custom python scripts were then used with the Entrez API to filter proteins that contain “regulator” in their annotation and that are adjacent to proteins annotated as “multidrug efflux pumps”. The resulting regulators were then used as inputs to generate operator predictions with Snowprint, and the 33 regulators with the top consensus scores were selected for functional testing within *E. coli* cells. The Snowprint logo was generated using a vector graphics editor.

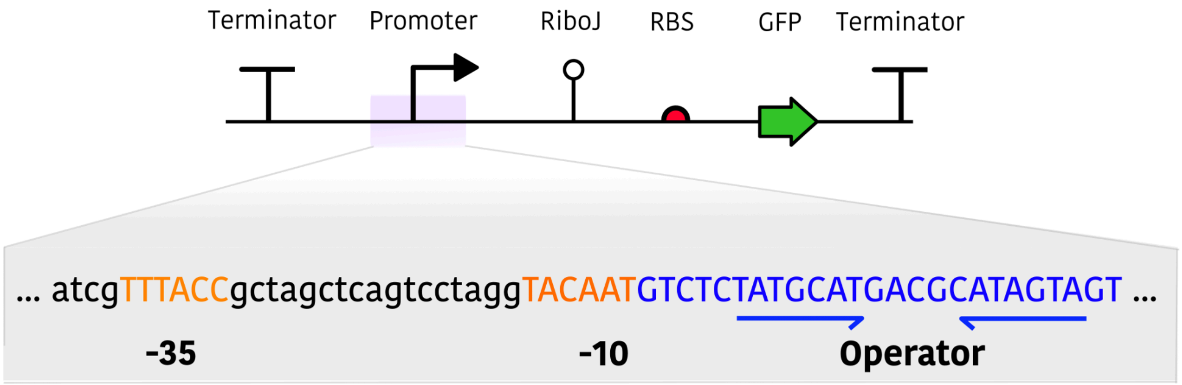

Supplementary Figure 5. Promoter design for domesticated regulators

Regulator-controlled promoters were designed by placing the predicted operator sequence immediately downstream from the -10 box of an E. coli sigma-70 promoter. Arrows indicate inverted repeat sequences, showing that five base pairs outside of the inverted repeat region were included in the promoter. The broader context of the reporter transcriptional unit is shown above the promoter sequence.

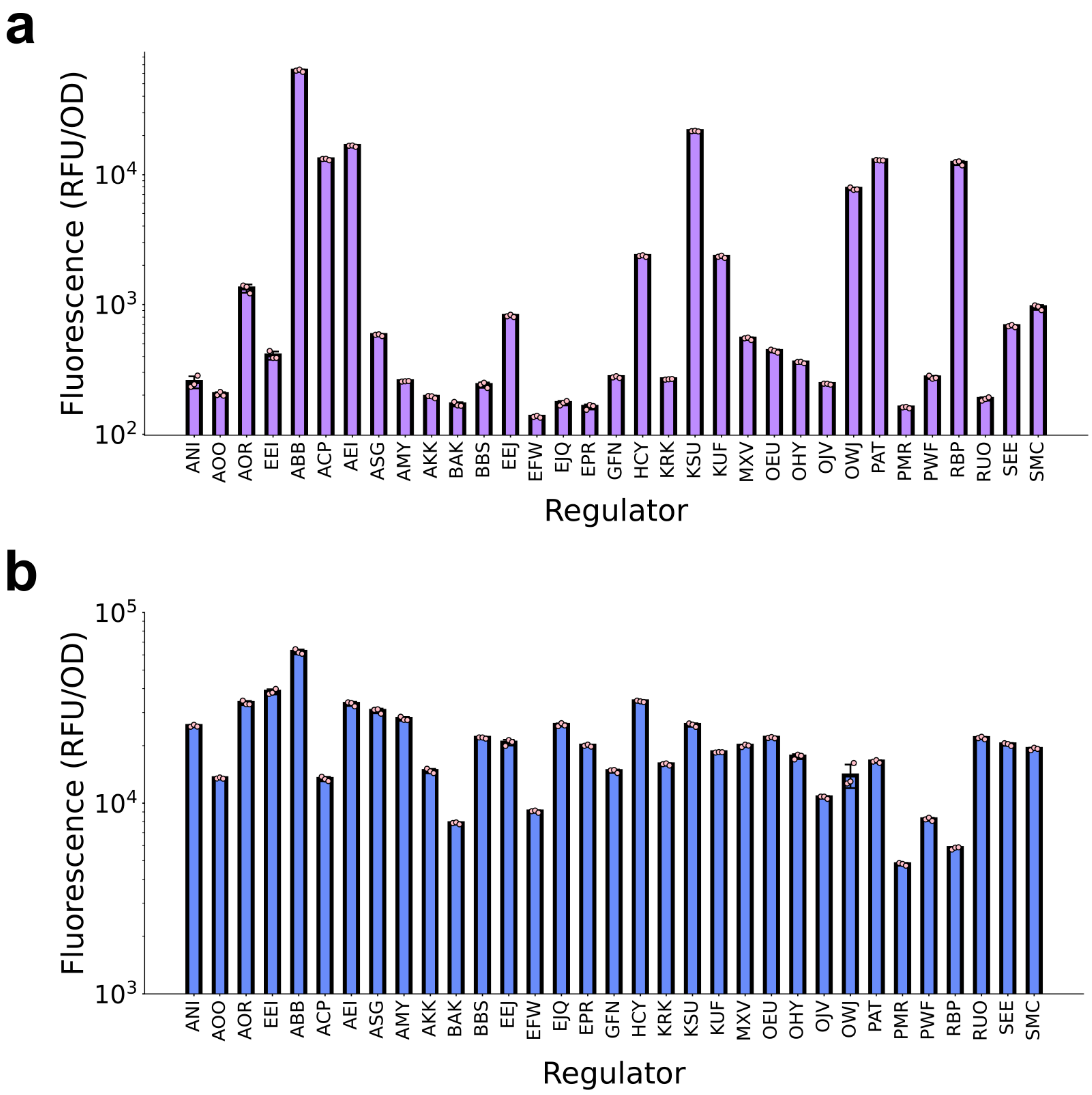

Supplementary Figure 6. Synthetic promoter output with and without domesticated regulator expression. Fluorescence of *E. coli* cells containing a reporter plasmid expressing GFP and a regulator plasmid expressing a transcription factor (see Figure 3, Supplementary Figure 10). The GFP-expressing promoter contains a Snowprint-predicted operator (see Supplementary Figure 5). In (a) a regulator plasmid expresses the transcription factor predicted to bind to the Snowprint-predicted operator. In (b) the regulator plasmid expresses the CamR transcription factor, which serves as a control transcription factor that should not bind to the promoter driving GFP expression. Assays were performed in biological triplicate. Individual data points are shown in pink. Error bars represent the standard deviation +/- the mean.

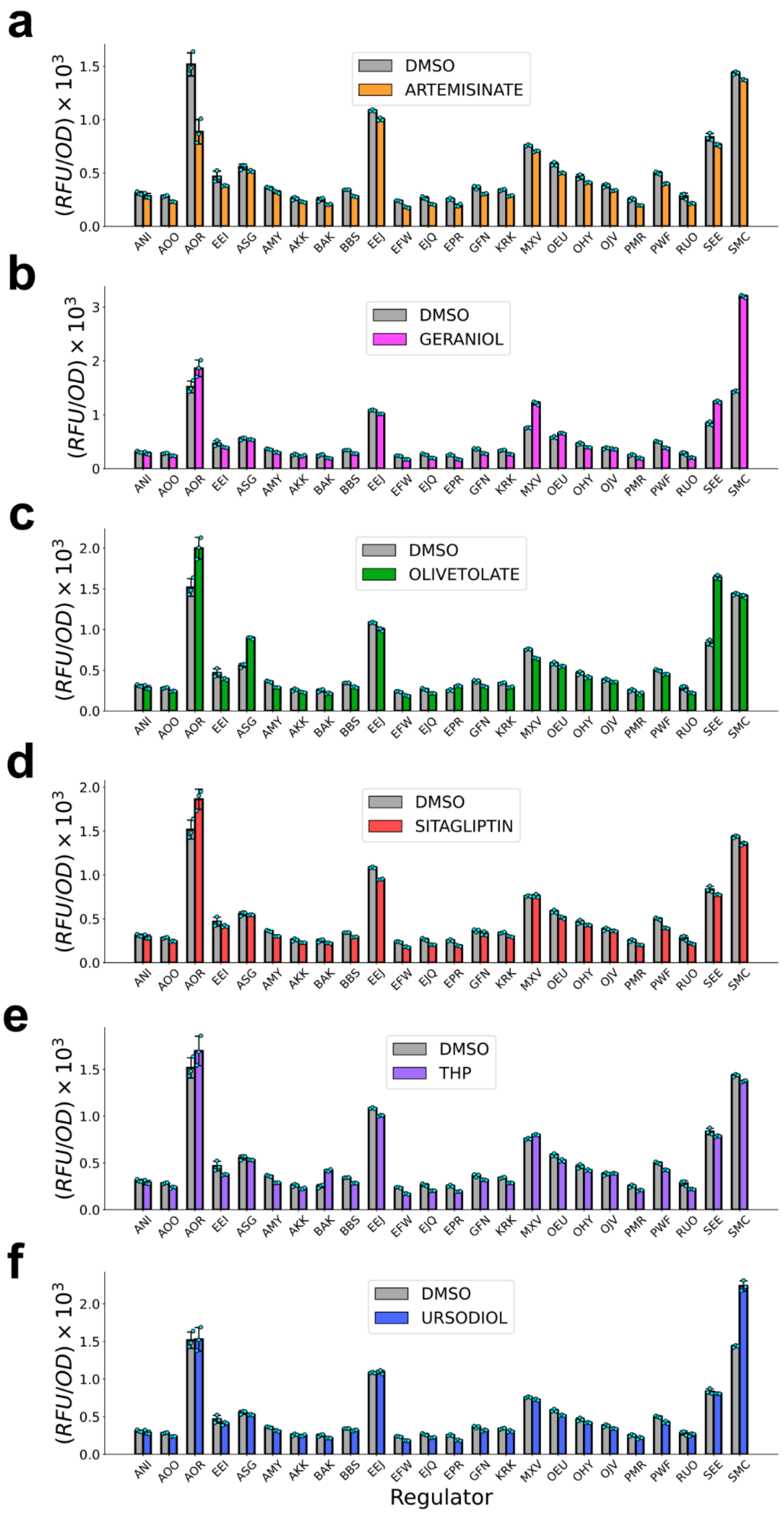

Supplementary Figure 7. Response of 24 generalist regulators to biomanufacturing-relevant ligands. The final concentration for induction of each ligand was 100 uM. Assays were performed in biological triplicate. Individual data points are shown in cyan. Error bars represent the standard deviation +/- the mean.

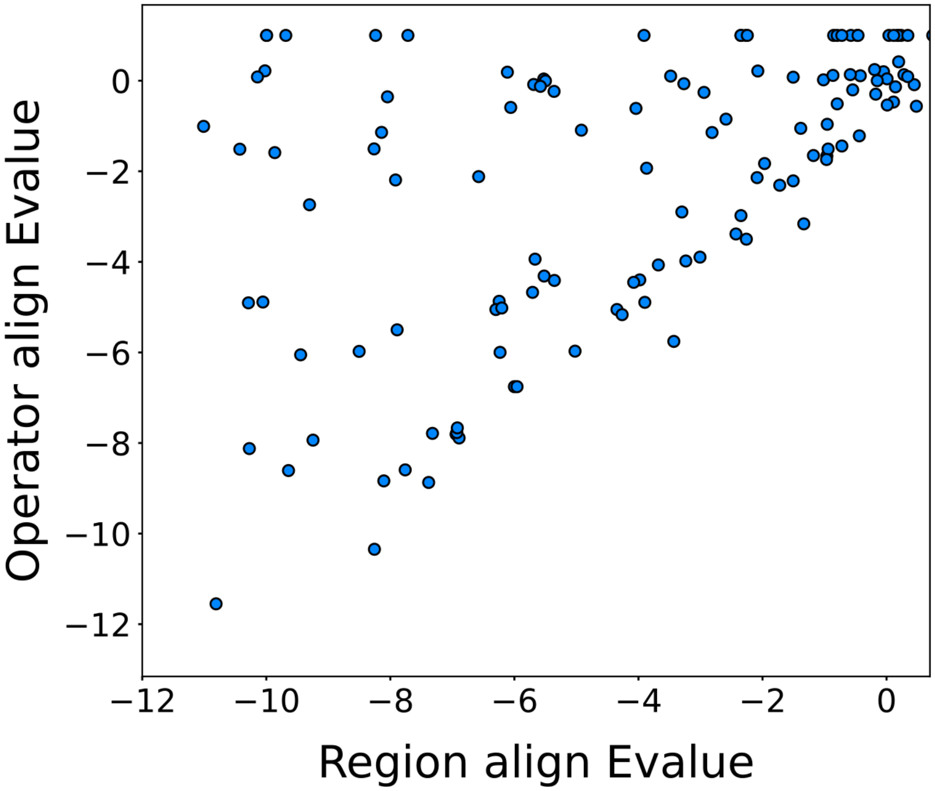

Supplementary Figure 8. Comparison of known operator alignment to the predicted operator vs the inter-operon region. Single data points represent the E-value similarity score of a known operator for its corresponding Snowprint predicted operator and the inter-operon region within the host organism's genome predicted to contain the operator.

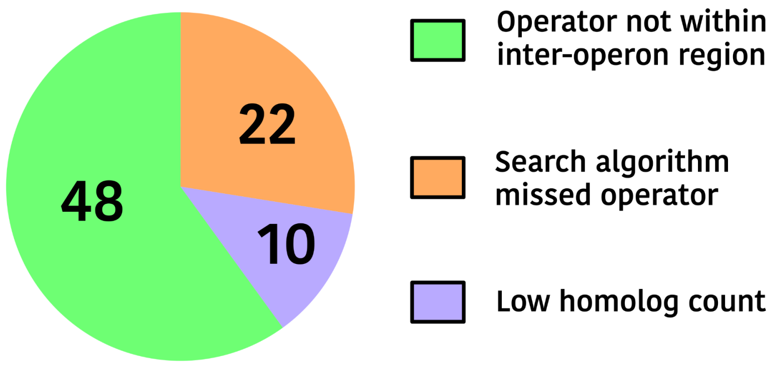

Supplementary Figure 9. Frequencies of failure modes encountered during benchmarking

Snowprint failure modes were binned into three categories: (1) the operator is not found within the inter-operon region, (2) the search algorithm failed to identify the validated operator, and (3) the prediction quality is low due to fewer than 10 homologs being used to create the motif. To be binned into category (1), the validated operator must not align to the extracted inter-operon region (alignment score lower than 90/100). To be binned into category (2), the validated operator must align nearly perfectly to the inter-operon region (alignment score of >90/100), but align poorly to the predicted operator (alignment score of <60/100). All benchmarking metrics used to create this plot can be found in **Supplementary Data 1.**

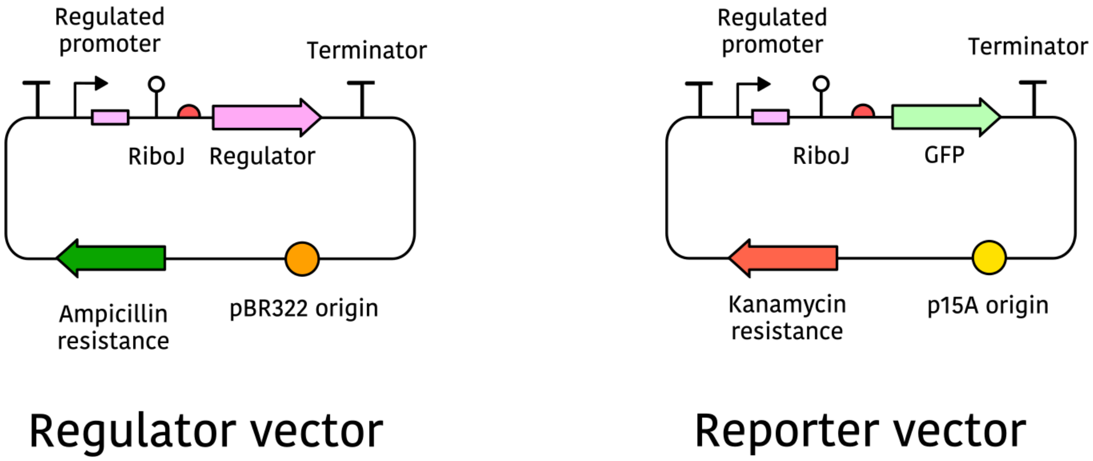

Supplementary Figure 10. Schematics of the two circuits architectures used in this study

The pink regulator gene and regulated promoter sequences change for each regulator being tested. Please refer to **Supplementary Figure 5** for an in-depth illustration of the Regulated Promoter region. Please refer to, **Supplementary Table 2** for the GenBank IDs and predicted operator sequences of all regulators. Please refer to **Supplementary Table 3 & 4** for the full plasmid sequence of the Reporter vector and Regulator vector, respectively.

TGCTTCATTCCTCGGTACCAAATTCCAGAAAAGAGGGGAGCGGAAACCGCTCCCCTTTTTCGTTTTGGTCCCTCGC
TGCAGTGTCGTAAAAACCAGTTGGGGAGACGCTGTGAATCCCGGGCAAGCAAAATCGCTGGAAGTTCCTATACTTTC
TAGAGAATAGGAACTTCTTTCTAAATACATTCAAATATGTATCCGCTCATGAGACAATAACCCTGATAAATGCTTCAA
TAATATTGAAAAAGGAAGAGTATGAGCCATATTCAACGGGAAACGTCTTGCTCCAGGCCGCGATTAAATTCCAACAT
GGATGCTGATTTATATGGGTATAAATGGGCTCGCGATAATGTCGGGCAATCAGGTGCGACAATCTATCGATTGTATGG
GAAGCCCGATGCGCCAGAGTTGTTTCTGAAACATGGCAAAGGTAGCGTTGCCAATGATGTTACAGATGAGATGGTCA
GACTAAACTGGCTGACGGAATTTATGCCTCTTCCGACCATCAAGCATTTTATCCGTACTCCTGATGATGCATGGTTAC
TCACCACTGCGATCCCCGGGAAAACAGCATTCCAGGTATTAGAAGAATATCCTGATTCAGGTGAAAATATTGTTGATG
CGCTGGCAGTGTTCCTGCGCCGGTTGCATTCGATTCCTGTTTGTAATTGTCCTTTTAACAGCGATCGCGTATTTCGCC
TCGCTCAGGCGCAATCACGAATGAATAACGGTTTGGTTGATGCGAGTGATTTTGATGACGAGCGTAATGGCTGGCCT
GTTGAACAAGTCTGGAAAGAAATGCATAAGCTTTTGCCATTCTCACCGGATTCAGTCGTCACTCATGGTGATTTCTC
ACTTGATAACCTTATTTTTGACGAGGGGAAATTAATAGGTTGTATTGATGTTGGACGAGTCGGAATCGCAGACCGATA
CCAGGATCTTGCCATCCTATGGAACTGCCTCGGTGAGTTTTCTCCTTCATTACAGAAACGGCTTTTTCAAAAATATGG
TATTGATAATCCTGATATGAATAAATTGCAGTTTCATTTGATGCTCGATGAGTTTTTCTAAGTTGTGATGGCGGTAGG
AATGTAATCGTTAATCCGCAAATAACGTAAAAACCCGCTTCGGCGGGTTTTTTTATGGGGGGAGTTTAGGGAAAGAG
CATTTGTCATCCCGTTGAATATGGCTCCCTTAACGTGAGGAAGTTCCTATACTTTCTAGAGAATAGGAACTTCTACAG
ATGGACTTGGGTTGGCGGTTTCAGGAGTAGGTGCTTCCTCGCTCACTGACTCGCTGCACGAGGCAGACCTCAGCGCT
AGCGGAGTGTATACTGGCTTACTATGTTGGCACTGATGAGGGTGTCAGTGAAGTGCTTCATGTGGCAGGAGAAAAAA
GGCTGCACCGGTGCGTCAGCAGAATATGTGATACAGGATATATTCCGCTTCCTCGCTCACTGACTCGCTACGCTCGGTC
GTTCGACTGCGGCGAGCGGAAATGGCTTACGAACGGGGCGGAGATTTCCTGGAAGATGCCAGGAAGATACTTAACAG
GGAAGTGAGAGGGCCGCGGCAAAGCCGTTTTTCCATAGGCTCCGCCCCCCTGACAAGCATCACGAAATCTGACGCTCA
AATCAGTGGTGGCGAAACCCGACAGGACTATAAAGATACCAGGCGTTTCCCCCTGGCGGCTCCCTCGTGCGCTCTCCT
GTTCCTGCCTTTCGGTTTACCGGTGTCATTCCGCTGTTATGGCCGCGTTTGTCTCATTCCACGCCTGACACTCAGTTC
CGGGTAGGCAGTTCGCTCCAAGCTGGACTGTATGCACGAACCCCCCGTTCAGTCCGACCGCTGCGCCTTATCCGGTAA
CTATCGTCTTGAGTCCAACCCGGAAAGACATGCAAAAGCACCACTGGCAGCAGCCACTGGTAATTGATTTAGAGGAG
TTAGTCTTGAAGTCATGCGCCGGTTAAGGCTAAACTGAAAGGACAAGTTTTGGTGACTGCGCTCCTCCAAGCCAGTT
ACCTCGGTTCAAAGAGTTGGTAGCTCAGAGAACCTTCGAAAAACCGCCCTGCAAGGCGGTTTTTTCGTTTTCAGAGC
AAGAGATTACGCGCAGACCAAAACGATCTCAAGAAGATCATCTTATTAAGGGGTCTGACGCTCAGTGGAACGAAACC
CTGTTGGTCAGGTTTTCGGGAGGTGTGCGTCTCGCATCCGGAAAGGTGTGATAGGTAGCGCAGCAAATAAAACGAAA
GGCTCAGTCGAAAGACTGGGCCTTTCGTTTTATCTGTTGTTTGTCGGTGAACGCTCTCCTCAACGAAAATATATTTTT
CAAAAGTATCGTTTACCGCTAGCTCAGTCCTAGGTACAATNNNNNNNNNNNNNNNNNTGGCAGCTGTCACCGGAT
GTGCTTTCCGGTCTGATGAGTCCGTGAGGACGAAACAGCCTCTACAAATAATTTTGTTTAAGGGCCCAAGTTCACTTA
AAAAGGAGATCAACAATGAAAGCAATTTTCGTACTGAAACATCTTAATCATGCGGTAAGGAGTTAAATATGTCGAAG
GGAGAGGAATTGTTTACTGGAGTCGTCCCAATTCTTGTTGAGTTGGACGGAGATGTCAATGGTCATAAGTTTAGCGT
ATCCGGTGAGGGTGAGGGAGATGCTACTTACGGAAAATTAACATTAAAATTCATTTGTACCACAGGCAAACTTCCTGT
TCCTTGGCCAACTCTTGTGACGACTTTTGGCTACGGTGTGCAGTGTTTCGCTCGTTATCCTGACCACATGAAGCAACA
TGATTTCTTCAAGTCTGCGATGCCTGAAGGATATGTTCAAGAACGTACCATCTTCTTCAAAGATGATGGTAACTATAA
GACTCGTGCAGAGGTAAAATTCGAGGGAGATACGTTGGTAAATCGCATTGAGCTGAAGGGAATCGATTTCAAGGAAG
ATGGAAACATTCTGGGACACAAGCTGGAGTACAATTACAATAGCCATAACGTCTATATCATGGCAGATAAGCAAAAAA
ACGGAATCAAGGTTAACTTCAAGATTCGCCATAATATTGAGGACGGCTCTGTGCAATTGGCGGATCATTATCAACAGA
ACACCCCGATTGGAGATGGTCCCGTGCTGCTGCCAGATAATCACTATTTGTCTACACAATCGGCCCTTTCCAAAGATC
CGAACGAAAAGCGCGATCATATGGTACTTTTAGAGTTCGTAACTGCCGCTGGAATCACCCACGGCATGGATGAGTTGT
ATAAGTAATAATCC

p15A origin | Kanamycin resistance gene
GFP | Promoter
Predicted operator | RiboJ | Terminator

Supplementary Figure 11. Full plasmid sequence of the reporter vector

AAAACTCACGTTAAGGCCCTCTCCAAGACCGAGCCATCAACAAAGCGTCTCGCTGAGGTTTCATGGAGCCTCTGGTTCATCTCCGG
CAATTAAAAAAGCGGCTAACCACGCCGCTTTTTTTACGTCTGCAGGAACGGGCTGTCGACCTTTGAAAAGTTCGTTTACCGCTAGC
TCAGTCCTAGGTACAATNNNNNNNNNNNNNNNNNNNTGGCAGCTGTCACCGGATGTGCTTTCCGGTCTGATGAGTCCGTGAGGAC
GAAACAGCCTCTACAAATAATTTTGTTTAAGGGCCCAAGTTCACTTAAAAAGGAGATCAACAATGAAAGCAATTTTCGTACTGAAA
CATCTTAATCATGCGGAGGCAGCTTAAATATGTCTACTAAGAACAAGATTGACAAGAACTATCTGATCAACATCATCGAGGAGATCC
TGCTGAACGATGGAATTAGTGGCTTGAGCATTCGTAAAGTAGCCACTAAAGCTAATATCTCAATCGGTGGTGTTCAGTATATCTTCG
GCAATAAGGAAGGGATGATCAAAGCGGTCCTTGAAAAAAACGAAGAGGATTACAACCGCCAGATTAAAATTCTCCTGAAGGATGA
CAATAGCAAATACTCCCAGTTGAAGGCGCATATCGAGTATATTTCGAAGCACAACGATAATGAGGAGTTCGACAAAATCTCGAAGA
TTATTACGATTCTGCTCCAAGAGAAATTCGTGTTCGAAGGTCTGCAAGATTGGTACAGTACGTCTTTAAATAGCATTGACACCAATA
CCGATGAGGGCAAAAAATTGCGGCTGGCATTTCTGTTCTCCGAAGCCATGTTTACACTGATGACCTTAAAGTATATCAACATTAGCC
CGAAGGAACAGAAGGAGATTTTCGAAGATCTGAAAACCTTTTTACTTTAATAATCCTATCGCCACTTTCAGCCAAAAAACTTAAGA
CCGCCGGTCTTGTCCACTACCTTGCAGTAATGCGGTGGACAGGATCGGCGGTTTTCTTTTCTCTTCTCAACACCCTTCGCGTCAACA
CTTTTCCGCCAAGGAGACGGTTGGTCAGGTTTTCGGGAGGTGTGGCTGGAAGTTCCTATACTTTCTAGAGAATAGGAACTTCTTTC
TAAATACATTCAAATATGTATCCGCTCATGAGACAATAACCCTGATAAATGCTTCAATAATATTGAAAAAGGAAGAGTATGAGTATT
CAACATTTCCGTGTCGCCCTTATTCCCTTTTTTGCGGCATTTTGCCTTCCTGTTTTTGCTCACCCAGAAACGCTGGTGAAAGTAAA
AGATGCTGAAGATCAGTTGGGTGCACGAGTGGGTTACATCGAACTGGATCTCAACAGCGGTAAGATCCTTGAGAGTTTTCGCCCCG
AAGAACGTTTTCCAATGATGAGCACTTTTAAAGTTCTGCTATGTGGCGCGGTATTATCCCGTGTTGACGCCGGGCAAGAGCAACTC
GGTCGCCGCATACACTATTCTCAGAATGACTTGGTTGAGTACTCACCAGTCACAGAAAAGCATCTTACGGATGGCATGACAGTAAG
AGAATTATGCAGTGCTGCCATAACCATGAGTGATAACACTGCGGCCAACTTACTTCTGACAACGATCGGAGGACCGAAGGAGCTAA
CCGCTTTTTTGCACAACATGGGGGATCATGTAACTCGCCTTGATCGTTGGGAACCGGAGCTGAATGAAGCCATACCAAACGACGAG
CGTGACACCACGATGCCTGCAGCAATGGCAACAACGTTGCGCAAACTATTAACTGGCGAACTACTTACTCTAGCTTCCCGGCAACA
ATTAATAGACTGGATGGAGGCGGATAAAGTTGCAGGACCACTTCTGCGCTCGGCCCTTCCGGCTGGCTGGTTTATTGCTGATAAATC
TGGAGCCGGTGAGCGTGGATCGCGCGGTATCATTGCAGCACTGGGGCCAGATGGTAAGCCCTCCCGTATCGTAGTTATCTACACGAC
GGGGAGTCAGGCAACTATGGATGAACGAAATAGACAGATCGCTGAGATAGGTGCCTCACTGATTAAGCATTGGTAAGTTGTGATGG
CGGTAGGAATGTAATCGTTAATCCGCAAATAACGTAAAAACCCGCTTCGGCGGGTTTTTTTATGGGGGGAGTTTAGGGAAAGAGCA
TTTGTCATCCCGTTGAATATGGCTCCCTTAACGTGAGGAAGTTCCTATACTTTCTAGAGAATAGGAACTTCTACAGATGGACTTGGG
TTGGCGGTTTCAGGAGTCTGCAAAACGTCTGCGACCTGAGCAACAACATGAATGGTCATCGGTTTCCGTGTTTCGTAAAGTCTGGA
AACGCGGAAGTCAGCGCCCTGCACCATTATGTTCCGGATCTGCATCGCAGGATGCTGCTGGCTACCCTGTGGAACACCTACATCTGT
ATTAACGAAGCGCTGGCATTGACCCTGAGTGATTTTTCTCTGGTCCCGCCGCATCCATACCGCCAGTTGTTTACCCTCACAACGTTC
CAGTAACCGGGCATGTTCATCATCAGTAACCCGTATCGTGAGCATCCTCTCTCGTTTCATCGGTATCATTACCCCCATGAACAGAAA
TCCCCCTTACACGGAGGCATCAGTGACCAAACAGGAAAAAACCGCCCTTAACATGGCCCGCTTTATCAGAAGCCAGACATTAACGC
TTCTGGAGAAACTCAACGAGCTGGACGCGGATGAACAGGCAGACATCTGTGAATCGCTTCACGACCACGCTGATGAGCTTTACCGC
AGCTGCCTCGCGCGTTTCGGTGATGACGGTGAAAACCTCTGACACATGCAGCTCCCGCAGACGGTCACAGCTTGTCTGTAAGCGGA
TGCCGGGAGCAGACAAGCCCGTCAGGGCGCGTCAGCGGGTGTTGGCGGGTGTCGGGGCGCAGCCATGACCCAGTCACGTAGCGATA
GCGGAGTGTATACTGGCTTAACTATGCGGCATCAGAGCAGATTGTACTGAGAGTGCACCGGTGTGAAATACCGCACAGATGCGTAAG
GAGAAAATACCGCATCAGGCGCTCTTCCGCTTCCTCGCTCACTGACTCGCTGCGCTCGGTCGTTCGGCTGCGGCGAGCGGTATCAGC
TCACTCAAAGGCGGTAATACGGTTATCCACAGAATCAGGGGATAACGCAGGAAAGAACATGTGAGCAAAAGGCCAGCAAAAGGCC
AGGAACCGTAAAAAGGCCGCGTTGCTGGCGTTTTTCCATAGGCTCCGCCCCCCTGACGAGCATCACAAAAATCGACGCTCAAGTCA
GAGGTGGCGAAACCCGACAGGACTATAAAGATACCAGGCGTTTCCCCCTGGAAGCTCCCTCGTGCGCTCTCCTGTTCCGACCCTGCC
GCTTACCGGATACCTGTCCGCCTTTCTCCCTTCGGGAAGCGTGGCGCTTTCTCATAGCTCACGCTGTAGGTATCTCAGTTCGGTGTA
GGTCGTTCGCTCCAAGCTGGGCTGTGTGCACGAACCCCCCGTTCAGCCCGACCGCTGCGCCTTATCCGGTAACTATCGTCTTGAGTC
CAACCCGGTAAGACACGACTTATCGCCACTGGCAGCAGCCACTGGTAACAGGATTAGCAGAGCGAGGTATGTAGGCGGTGCTACAG
AGTTCTTGAAGTGGTGGCCTAACTACGGCTACACTAGAAGGACAGTATTTGGTATCTGCGCTCTGCTGAAGCCAGTTACCTTCGGA
AAAAGAGTTGGTAGCTCTTGATCCGGCAAACAAACCACCGCTGGTAGCGGTGGTTTTTTTGTTTGCAAGCAGCAGATTACGCGCAG
AAAAAAAGGATCTCAAGAAGATCCTTTGATCTTTTCTACGGGGTCTGACGCTCAGTGGAACG

pBR322 + ROP origin | Ampicillin resistance gene
Regulator (BAK as example) | Promoter
Predicted operator | RiboJ | Terminator

Supplementary Figure 12. Full plasmid sequence of the regulator expression vector

Supplementary Tables

<table>
<tr><th>Protein ID</th><th>E-value</th><th>Alignment</th></tr>
<tr><td>WP_001224188.1</td><td>$3.7*10^{-13}$</td><td>Documented 1 gttttataATAAACGGAgagttaTCCGTTTGTcaa 35
||||||||||||.|||||||||||||||||
Predicted 1 -----ataatAAACGGATAGTTATCCGTTTgtcaa 30</td></tr>
<tr><td>AEM66515.1</td><td>0.0012</td><td>Documented 1 TAGCCACGTCTGGACAAAGTGAGAGATCGTGTCTAGACAACGCCACGGTT 50
||||||||||||||||||||
Predicted 1 ------------------------gatcgTGTCTAGACAacgcc------ 20</td></tr>
<tr><td>NP_415533.1</td><td>0.0076</td><td>Documented 1 TGTAAATTTTGACCATTTGGTCCACTTTTTTCT 33
.|||.||||.|..|..|||.
Predicted 1 -TTAACTTTTTAAAAACTGGC------------ 20</td></tr>
<tr><td>WP_011060270.1</td><td>0.0064</td><td>Documented 1 -----TCAAACAAGTGTTTGTCAGG--- 20
|.||||...|||| |||..
Predicted 1 aagccTGAAACGTATGTT--TCAaacaa 26</td></tr>
<tr><td>WP_005058758.1</td><td>0.0072</td><td>Documented 1 ---------CTTAACGCATGGCATGTGCGTTATG 25
|.|.|||||.|.|||.
Predicted 1 ggtagCGTGCGTTACGCACGtcata--------- 25</td></tr>
<tr><td>CAY46636.1</td><td>0.0049</td><td>Documented 1 ---GCT-----TGTATGTACAAGT-- 16
.|| |||||.|||||.|
Predicted 1 acgtcTGTATATGTATATACAaataa 26</td></tr>
<tr><td>NP_391277.1</td><td>0.0061</td><td>Documented 1 ---AAATTTGTCCGTATACATTTT-- 21
.||||.||.||||.|.||.|.
Predicted 1 atttaATTAGTACGTACAAATataga 26</td></tr>
<tr><td>NP_227848.1</td><td>0.0018</td><td>Documented 1 --AATTTCTTTCTGAG-GAAGATAGA 23
|..||||....|.| |||||..|
Predicted 1 agacaTTCTCAAAGTGAGAAgaatg- 25</td></tr>
<tr><td>WP_003084488.1</td><td>1.0</td><td>Documented 1 tttcTAG--AGTAaaTACTCTAaagt-- 24
||..| .|......|.|...|.|
Predicted 1 --tcacgGCGGGGCGCGCCCCGCactgt 26</td></tr>
</table>

Supplementary Table 1. Global alignment of documented and predicted operators.
The first and last alignments display excellent and poor scores for comparison, respectively. All other alignments have E-values between 0.01 and 0.001. The pairwise EMBOSS needle algorithm was used to create alignments with default parameters. Documented operators represent sequences experimentally validated to bind the corresponding protein regulator. Refer to Supplementary Data 1 for more information on sequence metrics and sources.

Genbank ID	Number of aligned sequences	Consensus Score	Predicted operator
ANI80042.1	7	94.99	gttcgAAAATAAACAATCTTGTTTCTTTTattca
AOO80349	12	89.099	cctgcTAAACTAAACGGTTCAGTTTAtttcg
AOR61801.1	10	62.821	tatacCCATAATTAATGACCCAGAAGTCATTAATTATGGtgtcc
EEI22781.1	38	95.314	ctcatAGTGACACTGTGTCACTcgatt
ABB11310.1	24	85.375	cgattTGCGTGCCCCGGGTGCCGGGGCACGCAtcctg
ACP22327	15	75.794	cttatTAGATTTCAATTGACATCTAtatat
AEI82798.1	38	71.203	cttTGCGGTCCcGGACCGCAtga
ASG24684.1	9	100	ggactGTACTGTATGATACAGTACgtaat
AMY71450	11	82.012	caatcACTGTACCGTCCAGACGGTAAAGTcaacc
AKK09602.1	24	68.741	acataTATTTCATCAACCGATGAAATAcggag
BAK71752.1	36	89.688	tttatAATACATACGTATGTATTtaata
BBS35964.1	8	71.611	agataAGCTAAACTTGAATAAAGATTCAAGTTTAGCTacttt
EEJ50829.1	21	76.158	ggtgtGGTGACAAGTTGTCACCggttg
EFW06302.1	70	72.686	cctttTTTAAATGAATATTTATTCATTCAAAttata
EJQ60916.1	99	92.352	attgtCTAACAGTGTTAGtttga
EPR37357.1	9	90.361	tccagAACTGACCGGTCAGTTtttga
GFN32285.1	9	85.012	acaatATGAATGAATCATTCATttcga
HCY59852	24	81.386	aaACCTAGTAAGTTACTAGGTga
KRK48889	12	96.543	aacggTTGTAAATTTAAATTACAAagagt
KSU66562.1	34	56.409	aaacaGGTGAGCGCCCGAGCATTATACTCGGGCGCTCACCcgttt
KUF06535.1	18	86.464	agaatATACCCATCGGTATctagg
MXV43606.1	21	82.634	ttagcGTGGAAACCGATTAGTTTCCACcccct
OEU66612	6	84.907	aacacTTGTTTAAAACAAtcgtt
OHV36883.1	13	87.706	ctactACCGTCCGTCCGGACGGTcggtc
OJV33711	28	84.969	gcaaaACGTGAGTGAACGATCACTCACGTactag
OWJ67207.1	100	45.685	cccgtCCGCCCCCGATTTCTCGGGGTCGGcgctg
PAT04833.1	12	95.26	cgcccCAAGGTATTACCTTGcacac
PMR76500	16	96.12	tgttgTTAACGCCGTTAAtatag
PWF24669	15	62.766	ctctaAAATTAGGTCGGTTAACCAACCTAATTTtatgc
RBP44292.1	3	86.607	tttacAGGTATGCAAGCAAGTACTTACTTGCATACCTgttgc
RUO21779.1	11	71.091	gaatcTCGGACTGACTAGTCCGTCCGAtggtt
SEE04737	41	76.365	attgaATTGCACCAGATAGTGCAATaggag
SMC09139	8	90.222	ccTTGTGAATgagATTCACAAaa

Supplementary Table 2. Snowprint predictions for mined regulators.
Results and metadata associated with Snowprint predictions of TetR-family transcriptional regulators targeted for testing within *E. coli*. Regulators that produced a >20-fold dynamic range in *E. coli* are highlighted green. Number of aligned sequences represents the number of homologous operator sequences used to generate the consensus. See **Methods** for consensus score calculation.

Supplementary Notes

Supplementary Note 1: Definitions

<u>Dynamic range</u>
We define the dynamic range as the fluorescent signal produced by the un-repressed promoter (without expression of the cognate regulator), divided by the fluorescent signal produced by the same promoter when repressed (with expression of the cognate regulator).

<u>Induction ratio</u>
We define the "induction ratio" as the highest fluorescent signal of the cell population upon induction with the target ligand divided by the basal fluorescence of the cell population without induction with any ligand.

Supplementary Note 2: Comparison to other prediction tools

While several other existing tools also predict a transcription factor's DNA binding sequence, these tools rarely use the same inputs as Snowprint, making direct comparison virtually impossible. Below we comment on several other existing tools that make similar predictions.

Microfootprinter[2]
Outdated software, no longer operational, and the source code is not available

DMINDA 2.0[3]
Incomparable input requirements*:* a set of promoter sequences in FASTA format

MP3[4]
Incomparable input requirements: a set of promoter sequences in FASTA format

DeepGRN[5]
Incomparable input requirements: ChIP-Seq and RNA-Seq datasets

CiiiDER[6]
Incomparable input requirements: a set of genome sequences

PhyloGibbs[7]
Incomparable input requirements: a set of promoter sequences in FASTA format

Morpheus[8]
Incomparable input requirements: a set of genome sequences

LASAGNA-Search[9]
Incomparable input requirements*:* a set of promoter sequences

KEGRU[10]

Incomparable input requirements: ChIP-Seq datasets
ProNA2020[11]
Predicts the region of a protein expected to bind to DNA, rather than the DNA sequence itself

Supplementary References

1. Pratt, H. & Weng, Z. LogoJS: a Javascript package for creating sequence logos and embedding them in web applications. *Bioinformatics* **36**, 3573–3575 (2020).
2. Neph, S. & Tompa, M. MicroFootPrinter: a tool for phylogenetic footprinting in prokaryotic genomes. *Nucleic Acids Res.* **34**, W366–W368 (2006).
3. Yang, J., Chen, X., McDermaid, A. & Ma, Q. DMINDA 2.0: integrated and systematic views of regulatory DNA motif identification and analyses. *Bioinformatics* **33**, 2586–2588 (2017).
4. Liu, B. *et al.* An integrative and applicable phylogenetic footprinting framework for cis-regulatory motifs identification in prokaryotic genomes. *BMC Genomics* **17**, 578 (2016).
5. Chen, C. *et al.* DeepGRN: prediction of transcription factor binding site across cell-types using attention-based deep neural networks. *BMC Bioinformatics* **22**, 38 (2021).
6. Gearing, L. J. *et al.* CiiiDER: A tool for predicting and analysing transcription factor binding sites. *PLOS ONE* **14**, e0215495 (2019).
7. Siddharthan, R., Siggia, E. D. & Nimwegen, E. van. PhyloGibbs: A Gibbs Sampling Motif Finder That Incorporates Phylogeny. *PLOS Comput. Biol.* **1**, e67 (2005).
8. Minguet, E. G., Segard, S., Charavay, C. & Parcy, F. MORPHEUS, a Webtool for Transcription Factor Binding Analysis Using Position Weight Matrices with Dependency. *PLOS ONE* **10**, e0135586 (2015).
9. Lee, C. & Huang, C.-H. LASAGNA-Search: an integrated web tool for transcription factor binding site search and visualization. *BioTechniques* **54**, 141–153 (2013).
10. Shen, Z., Bao, W. & Huang, D.-S. Recurrent Neural Network for Predicting Transcription Factor Binding Sites. *Sci. Rep.* **8**, 15270 (2018).
11. Qiu, J. *et al.* ProNA2020 predicts protein–DNA, protein–RNA, and protein–protein binding proteins and residues from sequence. *J. Mol. Biol.* **432**, 2428–2443 (2020).
