# Supplementary material for: Snowprint: a predictive tool for genetic biosensor discovery
Source: Commun Biol. 2024 Feb 9;7:163. doi: 10.1038/s42003-024-05849-8 (PMC10858194; doi:10.1038/s42003-024-05849-8)
Supplement: Supplementary file 3 — Description of Additional Supplementary Files [file 42003_2024_5849_MOESM3_ESM.pdf]

Description of Additional Supplementary Files

File name: Supplementary Data 1
Description: Experimentally validated regulator:operator pairs for benchmarking Snowprint.

File name: Supplementary Data 2
Description: Complete Snowprint benchmarking results and metrics.

File name: Supplementary Data 3
Description: The source data for Figures 2b, 3c, and 4a-e.
