# Supplementary material for: Snowprint: a predictive tool for genetic biosensor discovery
Source: Commun Biol. 2024 Feb 9;7:163. doi: 10.1038/s42003-024-05849-8 (PMC10858194; doi:10.1038/s42003-024-05849-8)
Supplement: Supplementary file 7 — Reporting Summary [file 42003_2024_5849_MOESM7_ESM.pdf]

nature portfolio

Corresponding author(s): Simon d'Oelsnitz

Last updated by author(s): Dec 29, 2023

Reporting Summary

Nature Portfolio wishes to improve the reproducibility of the work that we publish. This form provides structure for consistency and transparency in reporting. For further information on Nature Portfolio policies, see our Editorial Policies and the Editorial Policy Checklist.

Statistics

For all statistical analyses, confirm that the following items are present in the figure legend, table legend, main text, or Methods section.

n/a	Confirmed	
☐	☒	The exact sample size (*n*) for each experimental group/condition, given as a discrete number and unit of measurement
☐	☒	A statement on whether measurements were taken from distinct samples or whether the same sample was measured repeatedly
☐	☒	The statistical test(s) used AND whether they are one- or two-sided<br>*Only common tests should be described solely by name; describe more complex techniques in the Methods section.*
☐	☒	A description of all covariates tested
☒	☐	A description of any assumptions or corrections, such as tests of normality and adjustment for multiple comparisons
☐	☒	A full description of the statistical parameters including central tendency (e.g. means) or other basic estimates (e.g. regression coefficient) AND variation (e.g. standard deviation) or associated estimates of uncertainty (e.g. confidence intervals)
☒	☐	For null hypothesis testing, the test statistic (e.g. *F*, *t*, *r*) with confidence intervals, effect sizes, degrees of freedom and *P* value noted<br>*Give P values as exact values whenever suitable.*
☒	☐	For Bayesian analysis, information on the choice of priors and Markov chain Monte Carlo settings
☒	☐	For hierarchical and complex designs, identification of the appropriate level for tests and full reporting of outcomes
☒	☐	Estimates of effect sizes (e.g. Cohen's *d*, Pearson's *r*), indicating how they were calculated

Our web collection on statistics for biologists contains articles on many of the points above.

Software and code

Policy information about availability of computer code

Data collection	The Tecan Infinite M1000 plate reader was used to collect optical density and fluorescence measurements for all cell samples.
Data analysis	Python (version 3.8) was used for plotting data and estimating non-linear functions. Code used to generate bar plots and dose–response functions presented in this text is accessible at https://github.com/simonsnitz/plotting. Custom code which composes the command line tool, frontend web application, and backend web application presented in this paper are available at https://github.com/simonsnitz/Snowprint , https://github.com/simonsnitz/snowprint-ui , and https://github.com/simonsnitz/snowprint-backend, respectively. The Snowprint repository was deposited in Zenodo.

For manuscripts utilizing custom algorithms or software that are central to the research but not yet described in published literature, software must be made available to editors and reviewers. We strongly encourage code deposition in a community repository (e.g. GitHub). See the Nature Portfolio guidelines for submitting code & software for further information.

Data

Policy information about availability of data

All manuscripts must include a data availability statement. This statement should provide the following information, where applicable:

- Accession codes, unique identifiers, or web links for publicly available datasets
- A description of any restrictions on data availability
- For clinical datasets or third party data, please ensure that the statement adheres to our policy

The relevant data are available from the corresponding authors upon request. The source data for Figures 2b, 3c, 4a, 4b, 4c, 4d, and 4e are provided in Supplementary Data 3. NCBI RefSeq identifiers for all mined regulators in this study can be found in Supplementary Table 2. The GenBank ID for the CamR protein used as a control regulator is BAA03510.1.

Human research participants

Policy information about studies involving human research participants and Sex and Gender in Research.

Reporting on sex and gender	N/A
Population characteristics	N/A
Recruitment	N/A
Ethics oversight	N/A

Note that full information on the approval of the study protocol must also be provided in the manuscript.

Field-specific reporting

Please select the one below that is the best fit for your research. If you are not sure, read the appropriate sections before making your selection.

☒ Life sciences ☐ Behavioural & social sciences ☐ Ecological, evolutionary & environmental sciences

For a reference copy of the document with all sections, see nature.com/documents/nr-reporting-summary-flat.pdf

Life sciences study design

All studies must disclose on these points even when the disclosure is negative.

Sample size	Each experimental condition was performed in biological triplicate. This sample size is in line the previous work in the field.
Data exclusions	Data were not excluded
Replication	Each experimental condition was performed in biological triplicate. This sample size is in line the previous work in the field.
Randomization	Samples were not randomized. Varying concentrations of inducer molecules and substrates were used, as described in the Methods section. The same experimental conditions were used for all samples that were compared to each other to minimize variation. This is standard in the field.
Blinding	Investigators were not blinded to experimental conditions, because similar strategies were required to be applied in all experiments.

Reporting for specific materials, systems and methods

We require information from authors about some types of materials, experimental systems and methods used in many studies. Here, indicate whether each material, system or method listed is relevant to your study. If you are not sure if a list item applies to your research, read the appropriate section before selecting a response.

Materials & experimental systems

n/a	Involved in the study
☒	☐ Antibodies
☒	☐ Eukaryotic cell lines
☒	☐ Palaeontology and archaeology
☒	☐ Animals and other organisms
☒	☐ Clinical data
☒	☐ Dual use research of concern

Methods

n/a	Involved in the study
☒	☐ ChIP-seq
☒	☐ Flow cytometry
☒	☐ MRI-based neuroimaging
